# Influence of Polymer Relaxation Time on the Electrospinning Process: Numerical Investigation

**DOI:** 10.3390/polym9100501

**Published:** 2017-10-12

**Authors:** Siddharth Gadkari

**Affiliations:** 1 Department of Mechanical and Aerospace Engineering, Monash University, Clayton 3168, Australia; sid.gadkari@gmail.com; Tel.: +44-7549-246-785; 2 Department of Chemical Engineering, Indian Institute of Technology–Bombay, Mumbai 400076, India

**Keywords:** electrospinning, elasticity, conformation-dependent drag, self-concentration

## Abstract

“Electrospinnability”, or the ease with which a solution can be used to obtain bead-free uniform fibers, depends on a large number of parameters, including solution properties, process parameters and ambient conditions. In this study, the effect of the polymer relaxation time on electrospinning of dilute polymer solutions is investigated numerically. It is shown that elastic stresses (ES) increase exponentially with the Deborah number (De). For each polymer concentration there exists a critical De below which the ES are insufficient to overcome capillary stresses (CS) and lead to the formation of beaded fibers. However, above the critical De, there is a higher probability of the ES overcoming the CS and leading to the formation of uniform fibers. This analysis suggests the possibility of improved electrospinnability even with dilute polymer solutions, provided the relaxation time is sufficiently large. It is also found that changes in the drag coefficient due to change in the polymer conformation and self-concentration of polymer molecules would become significant for the electrospinning of polymer solutions with a longer relaxation time and high conductivity.

## 1. Introduction

**Figure 1:**
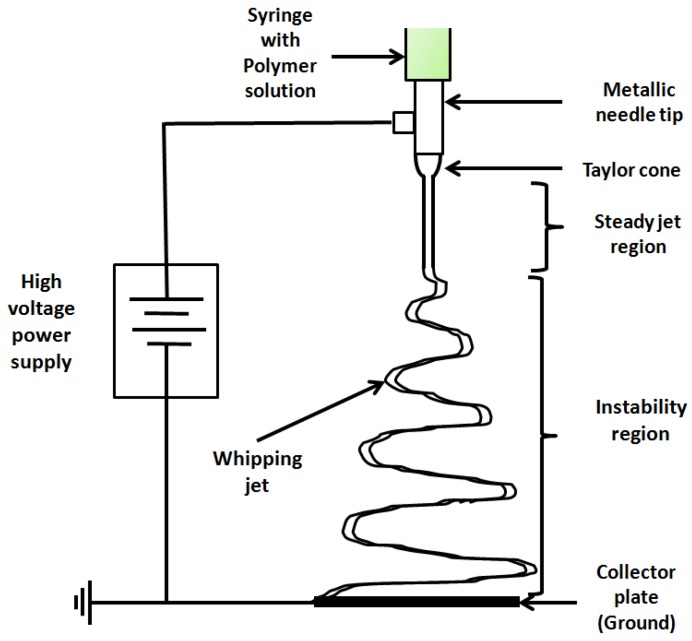
Typical experimental setup for electrospinning.

Electrospinning is a simple and versatile method to produce polymeric nanofibers. Such nanofibers possess properties such as a high surface-to-volume ratio, better surface functionality, a high degree of porosity, and so forth, which make them useful in a number of applications, such as wound dressings, drug delivery, filtration devices, protective clothing, fibers with specific surface chemistry and scaffolds useful in tissue engineering [1]. A typical electrospinning setup only requires a spinneret (syringe pump, syringe and a flat tip needle), a high voltage power supply and a collector plate, which is usually a conductor [1]. Figure 1 shows the basic schematic of the setup for electrospinning.

When a very high voltage, of the order of kilovolts, is applied between a capillary (or a syringe needle tip) containing a polymer solution and a grounded collector, the drop at the tip of the needle undergoes transformation into a conical shape, known as the “Taylor cone”. On increasing the voltage further, an electrified thin fluid jet is ejected from the tip of the Taylor cone. The jet is initiated only after the electrical forces at the surface overcome the surface tension and viscoelastic forces [2]. The jet follows a straight path for a certain distance but soon succumbs to numerous electrohydrodynamic instabilities, the most dominant being the whipping instability, which results in rapid chaotic movement of the jet in concentric circles of increasing diameter. This results in extensive elongation and thus extreme thinning of the jet. As the jet moves down, it dries and solidifies and is deposited on the collector [3].

While the electrospinning process is fairly straightforward to perform, not all polymer solutions can be electrospun. In fact, the “electrospinnability”, or the ease with which a solution can be electrospun to obtain bead-free uniform fibers, depends on a large number of parameters. Among others, these parameters include solution properties such as the polymer concentration, viscosity, conductivity and surface tension, as well as process parameters such as the applied voltage, solution flow rate, tip-to-collector distance, and so forth [1]. It has been observed that the final fiber diameter is related strongly to the polymer concentration in the precursor solution, and thinner fibers are obtained typically at a lower polymer concentration. A smaller diameter is desirable as it helps to realize the enhanced functionality of the fibers. However, decreasing the polymer concentration also drastically affects the electrospinnability of the polymer solutions. Electrospinning using dilute polymer solutions has been shown to result in beaded fibers or polymer droplets [4]. This is likely because the total elastic stresses (ES) generated in a dilute polymer solution are not high enough to compete with the capillary stresses (CS). High CS tend to break the jet into droplets. To produce bead-free uniform fibers, the ejecting jet has to be stable throughout the jet trajectory until it reaches the collector plate. High viscous or ES (shear and extensional) have been shown to overcome the Rayleigh–Plateau instability (that leads to the breakup of jets) induced by CS and impede the formation of beaded fibers or polymer droplets. However, the source of such high stresses can be different along the jet path from the capillary tip to the collector plate [5].

Increasing the concentration (and consequently viscosity) while lowering the surface tension favors the formation of bead-free and uniform fibers. There are a large number of studies which suggest a significant role of polymer chain entanglements (resulting in large viscoelastic stresses) in the formation of uniform fibers [4,6,7]. The boundary between the semi-dilute unentangled and semi-dilute entangled regimes is defined by the concentration $c_{e}$ at which significant overlap of the polymer chains topologically constrains the chain motion, causing entanglement couplings. Gupta et al. [4] and McKee et al. [6] showed that it is possible to obtain uniform bead-free fibers only in semi-dilute entangled solutions (i.e., for concentrations $>c_{e}$); at lower concentrations, polymer droplets (dilute regime) or beaded fibers (semi-dilute unentangled regime) are formed. Shenoy et al. [7] further emphasized the role of chain entanglements in polymer/good solvent systems and proposed that a minimum of at least one entanglement per polymer chain is required to achieve sufficiently high enough ES to damp the capillary instability and obtain fibrous structures. They also showed that stable uniform fibers are obtained above 2.5 entanglements per chain. On the other hand, there are studies that do not consider entanglements as a necessary condition for the stabilization of the jet and thus in the production of uniform fibers. These studies have proposed several different strategies to improve the electrospinnability of dilute polymer solutions that have no entanglements [5,8,9,10,11]. In particular, Yu et al. [5] showed that a strong elastic response can help to stabilize the jet, which can be achieved even for dilute polymer solutions if the polymer relaxation time is comparable to the extensional deformation of the jet. They showed that by increasing the relaxation time of the polymer solution and keeping the concentration constant, it is possible to improve the morphology of fibers from an initial beads-on-string structure at a shorter relaxation time to uniform fibers at a longer relaxation time. The concentrations they used were well below $c_{e}$. It was observed that the larger relaxation time imparts a high degree of elasticity that prevents the breakup of the jet into droplets. This stabilization mechanism can be understood by the growth of ES in the jet. Electrospun jets are subjected to a tensile pulling force due to the action of an external electric field and repulsion between like charges on the jet surface. If the time scale of the extensional deformation due to either of these electrostatic stresses is fast compared to the inverse of the solution relaxation time, this will lead to a build-up of ES in the fluid jet. If the ES are above a certain critical value, they can retard the capillary instability and thus delay the formation of polymer droplets or beads on string structures until solvent evaporation causes solidification of the polymer in a fiber.

In this work, a numerical analysis of the electrospinning process is performed to study the role of the relaxation time in the development of ES in the electrospinning of dilute polymer solutions. Using electrohydrodynamic equations valid for the steady jet region of electrospinning, the effect of the Deborah number (De; ratio of polymer relaxation time to time scale of the process) on the development of ES along the jet path is studied, to qualitatively explain the results observed by Yu et al. [5]. The De gives an indication of the extent of the elasticity of a polymer solution. Additionally, as the jet undergoes strong extensional flow in electrospinning, the polymer molecules undergo a coil–stretch transition. This causes a large jump in ES and also leads to enhanced intermolecular hydrodynamic interactions (HI), which leads to the self-concentration of the dilute polymer solution, thus making the average frictional drag coefficient concentration-dependent. The simple FENE-P (finitely extensible nonlinear elastic-Peterlin) model assumes a constant drag coefficient and cannot predict self-concentration. In this work, a novel polymer constitutive model proposed by [12,13] (which will be called the “CDD-sc” model in this work) is used to account for the changes in the drag coefficient and self-concentration effects.

## 2. Model Formulation

The electrohydrodynamics for the complete electrospinning process describing both the steady jet and instability region is extremely complex. Thus far, continuum simulations have examined only the steady jet region of the electrospinning jet [14,15,16]. In this work too, only the steady jet region is considered, and the governing equations pertinent to this regime, namely, the conservation of mass, the conservation of charge, the equation of motion and the electric field balance, are solved. The polymer contribution is accounted for using the FENE-P and the CDD-sc models. The constitutive models are expressed in the polymer conformation formalism instead of polymer stress, which was used by Carroll and Joo [16].

### 2.1. Governing Equations

Currently there are two main types of numerical approaches that are used to model the jet profile during electrospinning. The first approach considers the jet as a charged continuum and predicts the jet behaviour by solving equations for continuum mechanics [16,17]. In the second approach, the jet is represented by a series of discrete charged beads connected by viscoelastic springs, and here the jet profile is predicted by solving the equations of Newtonian mechanics [2,18,19,20]. While the continuum model is limited to the steady jet region of the electrospinning, the discrete (bead-spring) model allows for the prediction of whipping instability as it is developed. However, the discrete approach fails to account for the stable jet in detail [21,22].

In this work, we are primarily concerned about tracking the development of elastic, viscous and CS once the fine jet is ejected from the Taylor cone. It is very important to follow the growth of these stresses in the stable jet region, as the value of stresses at the end of stable jet define how strongly the jet can retard the formation of beads on the fibers, depending on the relative magnitude of elastic and CS. Thus, although it cannot predict the whipping instability, we have used the continuum model in this numerical analysis because it provides a detailed description of the stable jet region.

The continuum model assumes that the jet will travel in a straight line from the needle tip to the collector plate; however, in real experiments, the jet will bend after a certain distance. The criterion for selecting the end point of the stable jet region is discussed in Section 2.4, after presenting the governing equations of the model.

On the basis of the continuum approach, the governing equations for the steady jet region (Equations (1)–(4)) describe the steady-state variation of the jet radius *R*, the axial jet velocity *v*, the axial component of the electric field *E* and the surface charge density σ with axial variable *z*.

As described above in the introduction section, the fine jet ejected from the Taylor cone travels in straight path for a short distance but soon succumbs to radial distortion (originating from whipping instability). The jet bends and starts to move almost horizontally in loops of increasing diameter, which leads to rapid stretching and a dramatic decrease in the jet diameter. The concentric loops of growing size and the simultaneously decreasing jet diameter facilitate the increased surface area with the surrounding air and the smaller diffusion length, thus providing exceeding conditions for solvent evaporation in the whipping zone and further leading to jet solidification [23]. Thus, it can be assumed that most of the evaporation takes place in the whipping region. Spectroscopic data (Raman spectrum) has also confirmed that the solvent evaporation in the stable jet region is negligible [24]. As the continuum model used in the current work is only limited to the stable jet region, solvent evaporation has not been considered in this analysis.

(1) $$\begin{array}{c} \pi R^{2}v=Q_{f} \end{array}$$

(2) $$\begin{array}{c} \pi R^{2}KE+2\pi Rv\sigma =I \end{array}$$

(3) $$\begin{array}{c} \rho v\frac{dv}{dz}=\rho g+\frac{3\;\eta_{s}}{R^{2}}\frac{d}{dz}\left(R^{2}\frac{dv}{dz}\right)+\frac{1}{R^{2}}\frac{d}{dz}\left[R^{2}\;\left(\tau_{pzz}-\tau_{prr}\right)\right]+\frac{\gamma }{R}\frac{dR}{dz}+\frac{\sigma }{\bar{\epsilon }}\frac{d\sigma }{dz}+\left(\epsilon -\bar{\epsilon }\right)E\frac{dE}{dz}+\frac{2\sigma E}{R} \end{array}$$

(4) $$\begin{array}{c} E\left(z\right)=E_{\infty }-\ln\chi \left(\frac{1}{\bar{\epsilon }}\frac{d}{dz}\left(\sigma R\right)-\frac{\beta }{2}\;\frac{d^{2}}{{dz}^{2}}\left(ER^{2}\right)\right) \end{array}$$

The normal polymer stress difference is expressed as the following (Equation (5)):

(5) $$\begin{array}{c} \tau_{pzz}-\tau_{prr}=-\frac{3nk_{B}T}{{\langle Q^{2}\rangle }_{0}}\bar{f}\left(M_{zz}-M_{rr}\right) \end{array}$$

where

(6) $$\begin{array}{c} \bar{f}=\frac{L^{2}-{\langle Q^{2}\rangle }_{0}}{L^{2}-\langle Q^{2}\rangle }\;\mathrm{for}\;\mathrm{both}\;\mathrm{the}\;\mathrm{FENE-P}\;\mathrm{and}\;\mathrm{CDD-sc}\;\mathrm{models} \end{array}$$

and *M* is the conformation tensor, which is a second-order tensor that characterizes the structure of the molecule and is defined as M=〈QQ〉. A detailed description of *M* and its components $M_{zz}$ and $M_{rr}$ can be found in the Supplementary Material. Subscripts zz and rr represent the normal components of the respective tensor in the cylindrical coordinate system.

The Eulerian steady-state equations for the polymer conformation tensor components are as follows:

(7) $$\begin{array}{c} v\frac{dM_{zz}}{dz}=2\;\frac{dv}{dz}\;M_{zz}-\frac{1}{\lambda_{0}}\;\frac{1}{\zeta /\zeta_{0}}\left[\bar{f}\;M_{zz}-\frac{{\langle Q^{2}\rangle }_{0}}{3}\right] \end{array}$$

(8) $$\begin{array}{c} v\frac{dM_{rr}}{dz}=-\;\frac{dv}{dz}\;M_{rr}-\frac{1}{\lambda_{0}}\;\frac{1}{\zeta /\zeta_{0}}\left[\bar{f}\;M_{rr}-\frac{{\langle Q^{2}\rangle }_{0}}{3}\right] \end{array}$$

The derivation of the polymer constitutive models in terms of polymer conformation are described in detail in the Supplementary Material. The governing equations are non-dimensionalized by selecting the following characteristic scales: $r_{0}$ for *R* and *z*, $v_{0}=Q_{f}/\left(\pi r_{0}^{2}\right)$ for velocity *v*, $E_{0}=I/\left(\pi r_{0}^{2}K\right)$ for electric field intensity *E*, $\sigma_{0}=\bar{\epsilon }E_{0}$ for surface charge density σ, and $M_{0}={\langle Q^{2}\rangle }_{0}$ for polymer conformation tensor components $M_{zz}$ and $M_{rr}$.

Using the above scaling, the following non-dimensional governing equations are obtained:

(9) $$\begin{array}{c} R^{2}\;v=1 \end{array}$$

(10) $$\begin{array}{c} E\;R^{2}+Pe\;R\;v\;\sigma =1 \end{array}$$

(11) $$\begin{array}{c} v\;v^{\prime }=\frac{1}{Fr}+\frac{3\;B}{Re}\;\frac{1}{R^{2}}{\left(R^{2}\;v^{\prime }\right)}^{\prime }-\frac{3\;(1-B)}{De\;Re}\;\frac{1}{R^{2}}{\left[R^{2}\;\Gamma \;\left(M_{zz}-M_{rr}\right)\right]}^{\prime } \end{array}$$

(12) $$\begin{array}{c} \;\;\;\;\;\;\;+\frac{R^{\prime }}{We\;R^{2}}+\epsilon_{E}\left(\sigma \;\sigma^{\prime }+\beta \;E\;E^{\prime }+\frac{2\;\sigma \;E}{R}\right) \end{array}$$

(13) $$\begin{array}{c} E=\Omega -\ln\left(\chi \right)\left[{\left(\sigma \;R\right)}^{\prime }-\frac{\beta \;{\left(ER^{2}\right)}^{\prime \prime }}{2}\right] \end{array}$$

(14) $$\begin{array}{c} v\;\frac{dM_{zz}}{dz}=2\;v^{\prime }\;M_{zz}-\frac{1}{De}\;\frac{1}{\zeta /\zeta_{0}}\left[\Gamma \;M_{zz}-\frac{1}{3}\right] \end{array}$$

(15) $$\begin{array}{c} v\;\frac{dM_{rr}}{dz}=-\;v^{\prime }\;M_{rr}-\frac{1}{De}\;\frac{1}{\zeta /\zeta_{0}}\left[\Gamma \;M_{rr}-\frac{1}{3}\right] \end{array}$$

The prime indicates a derivative with respect to *z*.

The dimensionless groups obtained are:

Dimensionless NumberExpressionFroude number, Fr$v_{0}^{2}/gr_{0}$Reynolds number, Re$\rho v_{0}r_{0}/\eta_{0}$Weber number, We$\rho \;v_{0}^{2}\;r_{0}/\gamma$Electric Peclet number, Pe$2\bar{\epsilon }v_{0}/Kr_{0}$Deborah number, De$\lambda_{0}v_{0}/r_{0}$Electrostatic force parameter, $\epsilon_{E}$$\bar{\epsilon }E_{0}^{2}/\rho v_{0}^{2}$Viscosity ratio, *B*$\eta_{s}/\eta_{0}$Dielectric constant ratio, β$\epsilon /(\bar{\epsilon }-1)$Electric field strength, Ω$E_{\infty }/E_{0}$

*B* can also be expressed as $B=1/(1+\phi_{0})$, where $\phi_{0}=\eta_{p,0}/\eta_{s}$ refers to the initial concentration of the polymer solution, and $\eta_{p,0}=\eta_{0}-\eta_{s}$ is the polymer contribution to the zero shear-rate viscosity of the solution; $\eta_{0}$ and $\eta_{s}$ refer to the zero shear-rate viscosity and solvent viscosity, respectively.

(16) $$\begin{array}{cc} \Gamma & =\frac{N_{k}/\alpha^{2}-1}{N_{k}/\alpha^{2}-\left(M_{zz}+2M_{rr}\right)/3}\;\mathrm{for}\;\mathrm{FENE-P}\;\mathrm{and}\;\mathrm{CDD-sc}\;\mathrm{models} \end{array}$$

The respective values of $\zeta /\zeta_{0}$ for the different models are described in detail in the Supplementary Material 1.

### 2.2. Boundary Conditions

From the above non-dimensional governing equations, *v* and σ can be eliminated by using Equations (9) and (10). What remains are two second-order (Ordinary Differential Equations) ODEs for *R* and *E* and two first-order ODEs for $M_{zz}$ and $M_{rr}$ each. To solve this system of ODEs, the following six boundary conditions are used [15,16].

- First Boundary Condition:
  At the nozzle entrance, that is, at z=0, the jet radius is equal to the radius of the capillary $r_{0}$, which has also been used as scaling for *R*. Thus
  (17) $$\begin{array}{c} R(z=0)=1 \end{array}$$
- Second Boundary Condition:
  Asymptotic analysis suggests that, at z=χ, the acceleration of the jet is mainly governed by the tangential traction of the electric field:
  (18) $$\begin{array}{c} vv^{\prime }=\epsilon_{E}\frac{2\;\sigma \;E}{R} \end{array}$$
  which leads to the following scaling for the radius, $R\left(z\right)\propto z^{-1/4}$, near the collector plate. This scaling is used to obtain the boundary condition for *R* at z=χ as
  (19) $$\begin{array}{c} R\left(z=\chi \right)+4\chi R^{\prime }\left(z=\chi \right)=0 \end{array}$$
  However, the asymptotic balance assumed is only feasible for Newtonian solutions. For polymeric solutions, the asymptotic balance at χ may differ as the polymer stresses may be very high compared to the inertial and electric stresses.
  One possibility to solve this issue is to define a condition for $R^{\prime }$ at z=0. In this case, solving the ODEs is an initial-value problem, and the solution evolves to the value at z=χ on its own. However, there is not enough insight currently to define an additional initial condition at z=0, and hence an initial value solver cannot be used. However, given the fact that the stresses only need to be calculated at the end of the steady jet region, which appears much before z=χ, it is assumed that the properties at $z=z_{\max}$ (end of steady jet region) are not significantly affected by the boundary condition in Equation (19) at $z=\chi >>z_{\max}$.
- Third Boundary Condition:
  Assuming that the charge takes some time to migrate to the surface of the jet, it has been argued that the surface charge density at the origin of the jet (z=0) is negligible or zero. Thus
  (20) $$\begin{array}{c} \sigma (z=0)=0 \end{array}$$
- Fourth Boundary Condition:
  By the time the jet reaches the collector plate, the electric field variations in the electric field due to surface charge density become negligible, and the electric field becomes equal to the applied electric field (Ω). Thus
  (21) $$\begin{array}{c} E(z=\chi )=\Omega \end{array}$$
- Fifth and Sixth Boundary Conditions:
  It is assumed that there has been no significant stretching of the polymer molecules before they leave the jet. Thus the polymer coils are in equilibrium at the origin of the jet, and therefore the normal polymer conformation terms are equal to their equilibrium value of 1/3. Thus
  (22) $$\begin{array}{c} M_{zz}\left(z=0\right)=1/3 \end{array}$$
  and
  (23) $$\begin{array}{c} M_{rr}\left(z=0\right)=1/3 \end{array}$$

### 2.3. Numerical Method

The governing equations, including six first-order nonlinear coupled ODEs, were solved numerically using the boundary value solver in MATLAB, bvp4c [25]. Along with the governing equations and the boundary conditions, the solver also needs good initial guesses at each solver step for all the variables. The initial guesses must also satisfy the boundary conditions. To obtain better solutions, the dimensionless numbers were entered in a continuation scheme so as to start with simpler problems and use these as initial guesses for the next set of parameters. To provide the first initial guess, the approach from reference [16] was followed here, and the analytical results from a gravity thinning jet were used while making sure the contribution of the electric field was small. Equations were solved for various sets of dimensionless parameters to obtain predictions for the radius and electric field profiles under different operating conditions. The numerical analysis was validated by comparing against previously reported results in literature [14,15,16], and excellent agreement was obtained (see Supplementary Material). Feng [14], Feng [15], and Carroll and Joo [16] proposed electrohydrodynamic models for the stretching of electrified Newtonian jets and non-Newtonian jets using the well-known Giesekus and Oldroyd-B constitutive models. These models are also limited only to the straight-jet region of the electrospinning jet, and thus the thinning profiles obtained from the current model could be readily compared with those obtained from [14,15,16] for the same set of parameters.

### 2.4. Typical Values of Dimensionless Numbers and $z_{\max}$

To study the effect of the relaxation time for different polymer concentrations, the values of $\phi_{0}$ and De were changed systematically while keeping the values of all the other dimensionless numbers constant, as follows:

Re = 0.001, We = 0.001, Fr = 0.001, Pe = 0.004, χ = 300, β = 2, $\epsilon_{E}$ = 10, $E_{\infty }$ = 1, $N_{k}$ = 5000, $N_{k_{ref}}$ = 5000, and $z_{ref}$ = 1.

These values are close to those used by Carroll and Joo [16] in their study on the electrospinning of PIB (polyisobutylene) Boger fluids. An important point, which was also highlighted by Carroll and Joo [16], is that, because of limitations of the model and the numerical method used, the simulations for high conductivity (or low Pe) solutions, such as PEO (polyethylene oxide)–water systems, run into trouble as a result of numerical errors. Therefore, the simulations in this analysis were limited to Pe = 0.004 for low-conductivity solutions.

There is currently no fixed criterion available for the *z* value at which the steady jet region ends and the whipping instability sets in. Yu et al. [5] reported that jet bending starts when the fiber radius reaches about 10–20 μm. However, this may be only valid for the specific process parameters used by Yu et al. [5]. Carroll and Joo [16] observed that the steady jet region reaches about 2.5–5 mm from the nozzle tip for both Newtonian and polymer solutions when the total distance between the tip and collector plate varies from 13–17 cm. Experimental results of Helgeson et al. [26] show that the whipping region starts at 2–2.5 mm from the nozzle tip when the total distance between the nozzle tip and collector tip is maintained at 10 cm.

In the present analysis, the value of stresses near the onset of whipping need to be compared. On the basis of the observations of Carroll and Joo [16], the value of $z_{\max}$ is fixed as 10 for χ = 300. This value may not be true for solutions with different parameters values, and the exact $z_{\max}$ for each system may differ. However in this analysis, only a fixed value close to the onset of whipping is required, at which the stress contributions can be compared, and thus the assumption of $z_{\max}$ = 10 is reasonable.

### 2.5. Individual Stress Contributions

**Figure 2:**
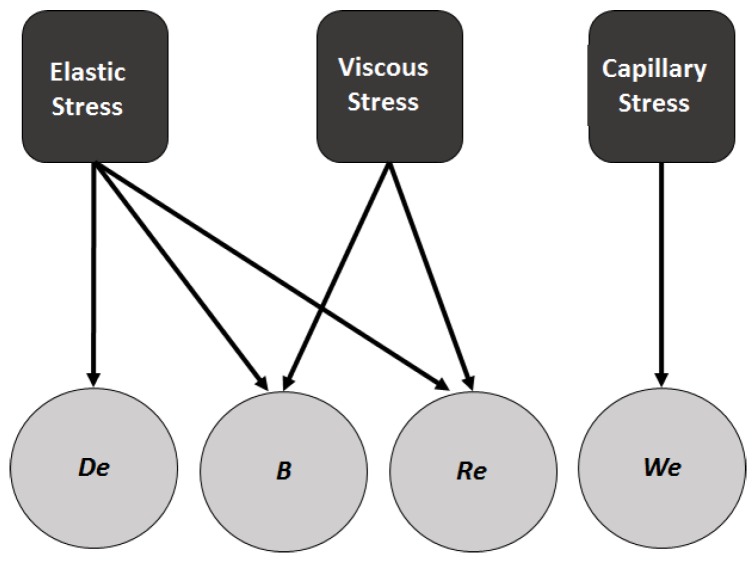
Relation between individual stress contributions and the dimensionless numbers.

The thinning profile data obtained from the simulations was used to calculate the capillary, viscous and elastic stress contributions, which are related to the dimensionless numbers We, *B*, Re and De, as shown in the schematic in Figure 2. The individual stress contributions are defined as follows:

(24) $$\begin{array}{c} \mathrm{Capillary}\;\mathrm{Stress:}\;\frac{1}{We}\;\frac{1}{R} \end{array}$$

(25) $$\begin{array}{c} \mathrm{Viscous}\;\mathrm{Stress:}\;\frac{3\;B}{Re}v^{\prime } \end{array}$$

(26) $$\begin{array}{c} \mathrm{Elastic}\;\mathrm{Stress:}\;\frac{\left(1-B\right)}{De\;Re}\left(\Gamma (M_{zz}-M_{rr})\right) \end{array}$$

Another quantity to be calculated is the local Weissenberg number, defined as

(27) $$\begin{array}{c} {\mathrm{Wi}}^{+}=De\frac{dv}{dz} \end{array}$$

## 3. Results and Discussion

### 3.1. Effect of Relaxation Time

**Figure 3:**
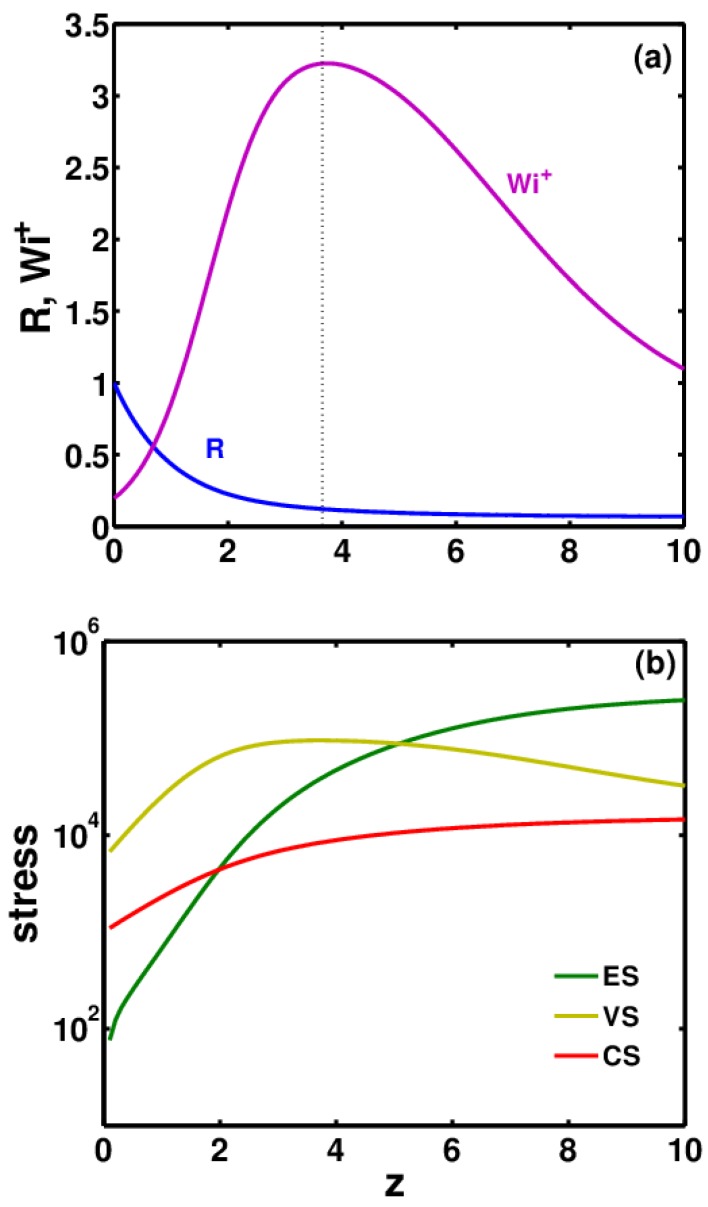
Typical profile of (**a**) jet radius *R* (blue curve) and local Weissenberg number ${\mathrm{Wi}}^{+}$ (pink curve) and (**b**) elastic (green curve), viscous (yellow curve) and capillary stresses (red curve) as function of *z* starting from nozzle tip to the end of steady jet region.

The simulation results provide *R*, *E*, $M_{zz}$ and $M_{rr}$ profiles as a function of distance *z*. A typical thinning profile is shown in Figure 3a for $\phi_{0}=0.02$ and De = 0.1. The jet first undergoes rapid thinning starting from the nozzle up to the Taylor cone region. The local strain rate or ${\mathrm{Wi}}^{+}$ increases rapidly in this region and grows beyond the critical value of 0.5 for the coil–stretch transition of polymer molecules. This fact is highly important for dilute polymer solutions, as no such transition can be observed in semi-dilute solutions. This transition can result in the formation of a percolated system of polymer macromolecules and in jet stabilization [27,28]. At the end of the Taylor cone, ${\mathrm{Wi}}^{+}$ reaches a maximum. The dotted line in Figure 3a represents the end of the Taylor cone. Beyond this, the viscous and ES had become significant enough to compete with the tensile pulling force exerted by electrical stresses. This slowed down the thinning considerably in the steady jet region, which caused ${\mathrm{Wi}}^{+}$ to decrease, although it remained above 0.5 (in some cases ${\mathrm{Wi}}^{+}$ can fall below 0.5). Hence ES continued to increase.

For models that cannot predict coil–stretch hysteresis (CSH), such as the FENE-P model, if ${\mathrm{Wi}}^{+}$ falls below 0.5, ES would begin to decrease. However, for models that can predict CSH, such as the CDD-sc model, a decrease in ES if ${\mathrm{Wi}}^{+}$ < 0.5 depends on the extent of the stretching initially. However, even in these models, if ${\mathrm{Wi}}^{+}$ decreases below the critical ${\mathrm{Wi}}^{+}$ for the stretch-to-coil transition, ES will relax.

With an increase in the thinning rate in the Taylor cone region, there is a huge increase in viscous stresses (VS), but thereafter they continue to decrease as the thinning rate slows down. In the steady jet region, VS begin to fall and the CS grow. Whether ES will grow or fall directly depends on the corresponding ${\mathrm{Wi}}^{+}$. If ES >> CS at the onset of whipping, it is possible that the ES would continue to dominate and the jet would remain stable in the whipping instability region. On the other hand, if CS >> ES, this could trigger the Rayleigh–Plateau instability leading to the formation of beaded fibers or polymer droplets.

**Figure 4:**
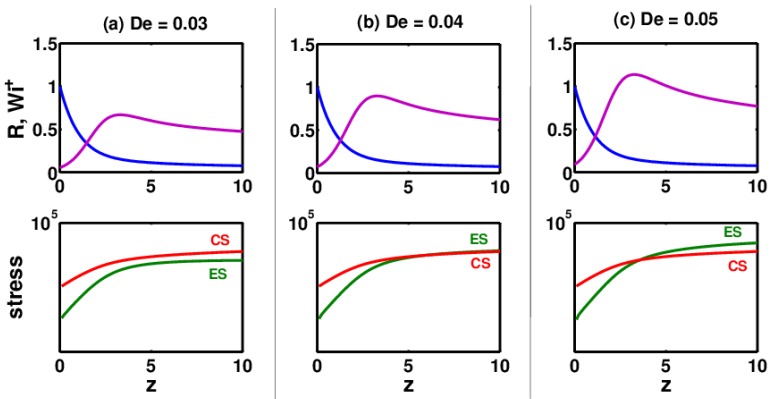
Growth of elastic stresses (ES) and capillary stresses (CS) as the jet begins to thin from the nozzle tip (*z* = 0) until the onset of whipping (*z* = 10) for three polymer solutions with constant $\phi_{0}=0.02$ but different De values: (**a**) De = 0.03, (**b**) De = 0.04 and (**c**) De = 0.05.

ES have been found to be a strong function of the relaxation time (or De) of the polymer solution. This is demonstrated in Figure 4, where three electrospinning solutions for the same set of parameters but different De numbers are compared. In Figure 4a, for De = 0.03, the ES stresses are smaller than the CS at $z=z_{\max}$. However, by increasing the De to 0.04 and keeping all the other parameters constant, the ES equal the CS at $z=z_{\max}$. With a further increase in the De number, the ES become much larger than the CS. The corresponding thinning radius (R) and ${\mathrm{Wi}}^{+}$ are also shown for the three values of the De number. Comparing these results with those obtained for De = 0.1 in Figure 3, it can be seen that the ES are significantly higher (>10${}^{5}$) than all the values in Figure 4. This again confirms the direct correlation between the De number and the ES.

**Figure 5:**
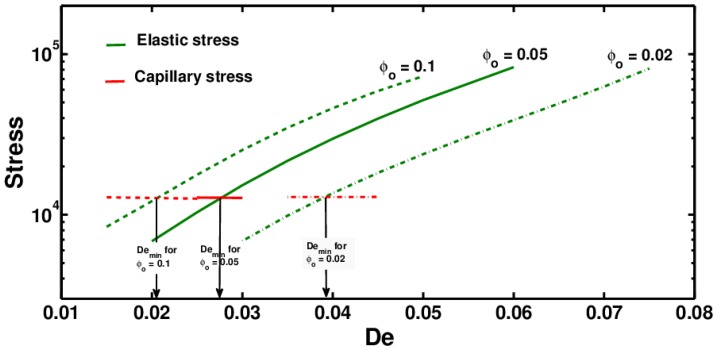
Elastic and capillary stresses at $z=z_{\max}$ as a function of De for polymer solutions with different initial concentrations.

At a fixed $z=z_{\max}$ with an increase in the De number, ES increase while CS remain almost constant. This is shown in Figure 5, which also shows the exponential increase in ES with an increase in De for polymer solutions with different initial concentrations: $\phi_{0}$ = 0.02, 0.05, 0.1.

Using these results, a minimum De ($De_{\min}$) is identified when the ES becomes equal to the CS. This $De_{\min}$ can serve as an indicator of whether the polymer solution will continue as a stable jet as it enters the whipping region.

**Figure 6:**
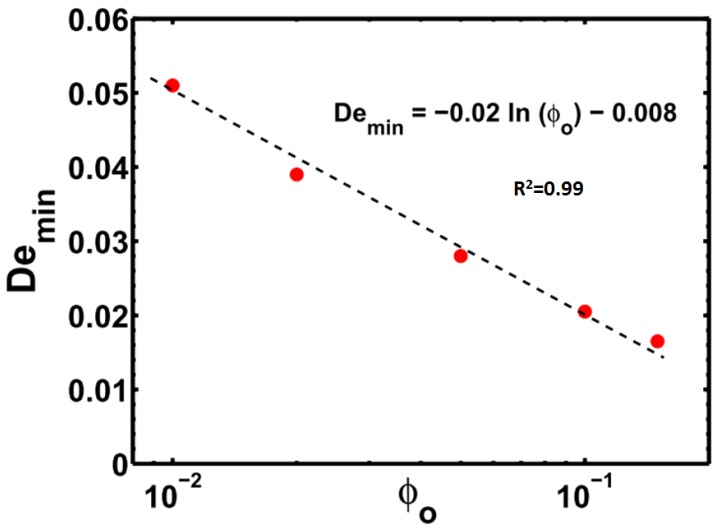
$De_{\min}$ as a function of polymer concentration $\phi_{0}$.

In Figure 5, $De_{\min}$ is the intersection point of the elastic and capillary stress curves. A polymer solution with $De<<De_{\min}$ has a higher probability of undergoing electrospraying in the instability region, and the jet may break up into small polymer droplets. The jet may still undergo whipping if the VS are high enough, but even such a jet would break up into droplets as the VS continued to fall and CS continued to grow along the length. If $De>>De_{\min}$, the ES would be large enough to suppress the Rayleigh–Plateau instability, and uniform fibers may be expected to form. $De_{\min}$ as expected is found to decrease with an increasing polymer concentration, as shown in Figure 5 and Figure 6.

As can also be seen from Figure 5, ES grow exponentially with an increase in De. This analysis shows that by increasing the De value of polymer solutions, it is possible to increase the ES and thus improve its electrospinnability.

### 3.2. Effect of Conformation-Dependent Drag and Self-Concentration

In dilute polymer solutions, the drag coefficient changes with change in the polymer conformation and also as the instantaneous pervaded volume fraction of the polymer solution changes due to self-concentration. However, the FENE-P model does not account for any of these changes and assumes a constant drag. A recently proposed “dumbbell” model [12,29] allows for these changes in the drag coefficient.

**Figure 7:**
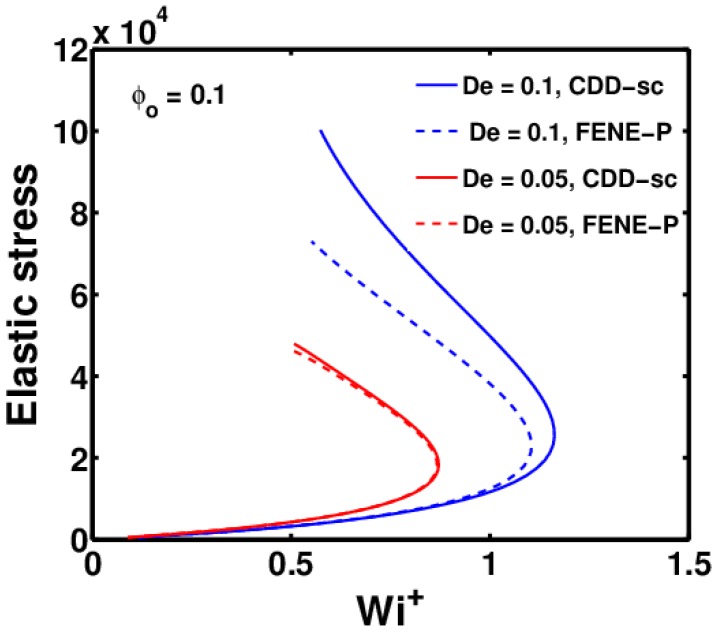
Variation of elastic stresses (ES) and ${\mathrm{Wi}}^{+}$ predictions for FENE-P and CDD-sc models, as the jet thins from nozzle tip up to $z_{\max}$, for $\phi_{0}$ = 0.1 at two different De numbers.

Figure 7 shows the variation in ES with ${\mathrm{Wi}}^{+}$ for z=0 to $z=z_{\max}$ using the CDD-sc and FENE-P models for two polymer solutions with a different relaxation time (De). As can be seen, there is almost no difference in ES obtained using the CDD-sc and FENE-P models for the polymer solution with a lower De number (De = 0.05). As De is increased to 0.1, ES at $z_{\max}$ obtained using the CDD-sc model are slightly higher than those obtained using the FENE-P model. This suggests that changes in the drag coefficient with conformation change and self-concentration become increasingly important with an increase in the De number.

**Figure 8:**
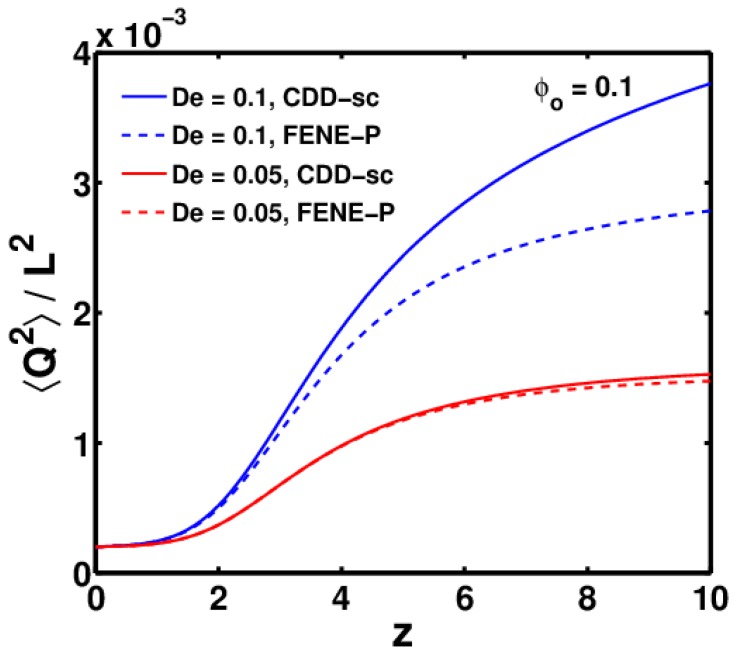
Variation of $\langle Q^{2}\rangle /L^{2}$ as a function of *z* for FENE-P and CDD-sc models, as the jet thins from nozzle tip up to $z_{\max}$, for $\phi_{0}$ = 0.1 at two different De numbers.

This is directly related to changes in the conformation of the polymer molecules, which can be measured using the ratio $\langle Q^{2}\rangle /L^{2}$, whose maximum value is 1, representing a completely stretched polymer molecule. Changes in the drag coefficient become significant only when the polymer molecules are sufficiently stretched. The relation between the two is explained in more detail in Figure 8, which shows the plot of $\langle Q^{2}\rangle /L^{2}$ as a function of *z* for the same parameters used in Figure 7.

As can be seen, the polymer solutions with De = 0.1 are stretched more compared to those with De = 0.05. Additionally, the difference between the FENE-P and CDD-sc models increases with the De number.

**Figure 9:**
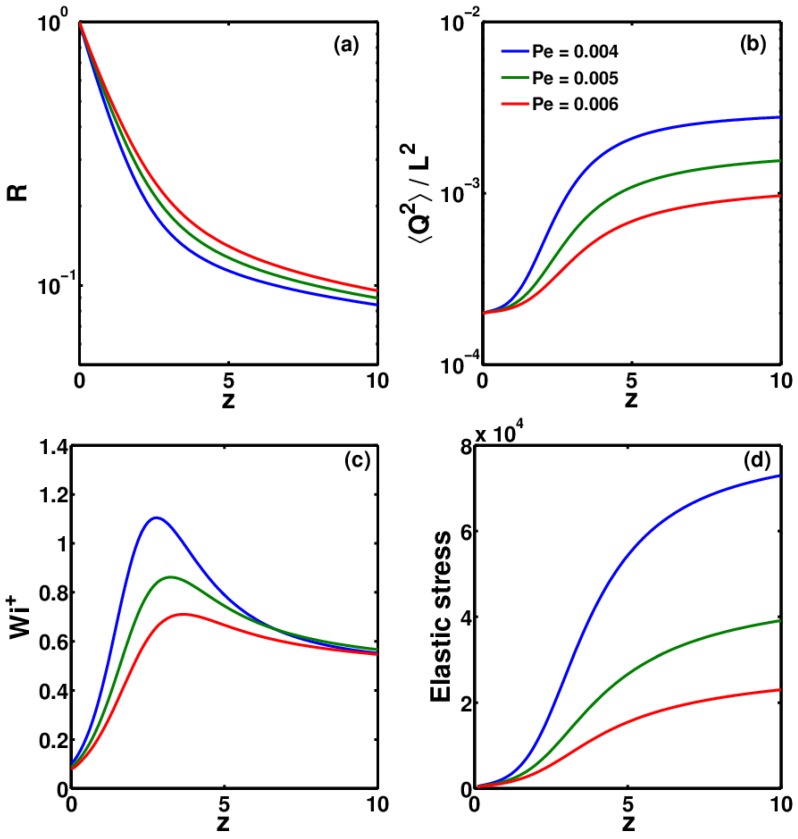
Variation of (**a**) R, (**b**) $\langle Q^{2}\rangle /L^{2}$, (**c**) ${\mathrm{Wi}}^{+}$ and (**d**) elastic stress as a function of *z*, for three polymer solutions with different Pe numbers.

Other than De, another major factor that governs the growth of ES is ${\mathrm{Wi}}^{+}$, which in turn depends on the rate of thinning. It has been observed that the highly conductive polymer solutions (such as PEO) thin at a much faster rate than low-conductivity polymer solutions (such as Boger fluids) [1]. Conductivity is reflected in the dimensionless electric Peclet number (Pe). A higher conductivity will result in low value of Pe and vice versa. In Figure 9, the effect of the Pe number on the thinning profile, $\langle Q^{2}\rangle /L^{2}$, and ES is shown. As can be seen, solutions with a higher Pe thin at a much faster rate in the Taylor cone, which leads to greater stretching of the polymer molecules in these solutions. The faster thinning rate induces a higher ${\mathrm{Wi}}^{+}$ number, which in turn leads to growth in ES. Therefore, for highly conductive polymer solutions, for which the Pe number is 3–4 orders of magnitude less than those used in this study, it is expected that the stretching would be much greater, which would lead to a significant difference between ES calculated using the CDD-sc and FENE-P models.

## 4. Conclusions

The experimental results of Yu et al. [5] have been numerically verified in this work in a qualitative way. It is shown that for each polymer concentration, there exists a critical De below which the ES are not sufficient enough to overcome the CS at the onset of whipping, and thus this may lead to beaded fibers or polymer droplets. However, above the critical De, the ES are dominant enough to maintain the stability of the jet as it enters the whipping region. Experimental researchers seeking to produce thin fibers from electrospinning can use dilute solutions of polymers with a high relaxation time. Given the operating parameters, a plot similar to Figure 6 can be generated to obtain the minimum relaxation time essential to obtain bead-free fibers at any given solution concentration. Beyond this minimum relaxation time, ES can arrest the break-up of the liquid jet or the formation of a beads-on-string structure and may lead to uniform fibers.

It should be noted that the stress balance (elastic vs. capillary) will change as the straight jet undergoes rapid stretching in the instability or whipping zone. Both ES and CS will increase in this region; however, it is assumed that the growth in ES would be much more significant because of the coil–stretch transition and the self-concentration of polymer molecules under the strong extensional flow. Thus, if the capillary stress is lower than the elastic stress at the onset of the instability region, it is expected that the high ES developed further on may provide a better probability of preventing bead formation as the jet whips down to the collector plate.

ES increase exponentially with an increase in the De number, and thus increasing the polymer relaxation time (or in effect the De number) can help to improve the electrospinnability of even dilute polymer solutions, as shown by Yu et al. [5] in their experimental study. Although it is found that there is no significant effect of self-concentration and CSH for the parameter values studied in this work, it is shown that these effects may become significant for polymer solutions with higher De numbers and a high conductivity (or a low Pe number). Additionally, as a future work, this study can be made more comprehensive by including the influence of external electric field intensity and the corresponding electric field stress on the fluid jet, in addition to the other stresses.

## Supplementary Materials

The following are available online at www.mdpi.com/2073-4360/9/10/501/s1.

## Nomenclature

**Parameter**	**Description**	**Unit**
*R*	Radius of the jet	m
$Q_{f}$	Applied flow rate	m${}^{3}$/s
*I*	Total current	A
$r_{0}$	Radius of the capillary or the needle tip	m
*v*	Fluid velocity parallel to axis of jet	m/s
*g*	Acceleration due to gravity	m/s${}^{2}$
*E*	Electric field	V/m
$E_{\infty }$	Applied electric field	V/m
*K*	Solution conductivity	S/m
*z*	Vertical distance along the jet	m
*L*	Total contour length of the polymer molecule	m
*n*	Number of polymer molecules per unit volume	1
$k_{B}$	Boltzmann’s constant	m${}^{2}$ kg s${}^{-2}$ K${}^{-1}$
Z	Solvent quality parameter	1
*T*	Absolute temperature	K
ϵ	Fluid dielectric constant	1
$\bar{\epsilon }$	Air dielectric constant	1
ρ	Solution density	kg/m${}^{3}$
α	Swelling ratio	1
$\phi_{0}$	Initial concentration of polymer solution	1
γ	Surface tension	N/m
σ	Surface charge density	c/m${}^{2}$
$\eta_{0}$	Zero shear-rate viscosity	N s/m${}^{2}$
$\eta_{s}$	Solvent viscosity	N s/m${}^{2}$
$\eta_{p,0}$	Polymer contribution to zero shear-rate viscosity	N s/m${}^{2}$
ζ	Mean drag coefficient of polymer molecule	1
$\zeta_{0}$	Equilibrium drag coefficient of polymer molecule	1
$\lambda_{0}$	Relaxation time of the polymer solution	s
χ	Aspect ratio	1
M	Conformation tensor	1
Q	End-to-end vector between the two beads	1
$\tau_{p}$	Polymer stress	N/m${}^{2}$
$N_{k}$	Number of Kuhn steps	1
〈〉	Angular brackets denote average values	−
${\langle Q^{2}\rangle }_{0}$	Mean squared end-to-end distance at equilibrium	m
$\langle Q^{2}\rangle /L^{2}$	Measure of change in conformation	1
*B*	Viscosity ratio	1
De	Deborah number	1
Re	Reynolds number	1
Fr	Froude number	1
We	Weber number	1
Pe	Electric Peclet number	1
$\epsilon_{E}$	Electrostatic force parameter	1
β	Dielectric constant ratio	1
Ω	Electric field strength	1
$Wi^{+}$	Weissenberg number	1
