# Supplementary material for: Influence of Polymer Relaxation Time on the Electrospinning Process: Numerical Investigation
Source: Polymers (Basel). 2017 Oct 12;9(10):501. doi: 10.3390/polym9100501 (PMC6418928; doi:10.3390/polym9100501)
Supplement: Supplementary file 1 [file polymers-09-00501-s001.zip › supportinginfo1.pdf]

Influence of polymer relaxation time on the electrospinning process: numerical investigation

Siddharth Gadkari

Supplementary Material 1: Derivation of Constitutive Models

Dumbbell models, characterized by two beads of identical mass joined by a connector, are the crudest representation of polymer molecules. These models in no way account for the details of the molecular architecture and do not have enough internal modes of motion to enable one to describe linear viscoelastic phenomena. However if the connector is represented by a spring, the so formed "elastic dumbbell model", is orientable and stretchable. These two properties are essential for the qualitative description of steady-state rheological properties and those involving slow changes with time.

The position and orientation of the dumbbell are specified by the position vectors of the centers of the two beads with respect to a laboratory-fixed coordinate system; these are designated $\boldsymbol{R}_1$ and $\boldsymbol{R}_2$, respectively. Then the configuration vector? or the "end-to-end vector", between the two beads is given by

$$\boldsymbol{Q} = \boldsymbol{R}_2 - \boldsymbol{R}_1 \tag{1}$$

The total flow induced stress tensor τ of the solution is made up of two parts, the solvent contribution ($\boldsymbol{\tau_s}$) and the polymer contribution ($\boldsymbol{\tau}_p$).

$$\boldsymbol{\tau} = \boldsymbol{\tau_s} + \boldsymbol{\tau_p} = \eta_s(\boldsymbol{\kappa} + \boldsymbol{\kappa}^T) + \boldsymbol{\tau_p} \tag{2}$$

The tensor $\boldsymbol{\kappa}$ is the transpose of the velocity gradient tensor, i.e. $\boldsymbol{\kappa} = (\nabla \boldsymbol{v})^{\boldsymbol{T}}$. The total contribution from polymer molecules (represented here by the dumbbells) comes in two parts, the "connector" contribution which originates from the tension in the connector springs and the "bead" contribution, which originates from the momentum transported by the bead. Adding the two contributions, we get the Kramers' general equation for polymer stress [1],

$$\boldsymbol{\tau_p} = \underbrace{n k_B T\, \boldsymbol{\delta}}_{\text{bead contribution}} - \underbrace{n\langle \boldsymbol{F^c}\, \boldsymbol{Q}\rangle}_{\text{connector contribution}} \tag{3}$$

The entropic force $\boldsymbol{F}^c$ is,

$$\boldsymbol{F^c} = H\, \boldsymbol{Q}\, f(\boldsymbol{Q}) \tag{4}$$

The function f will depend on the type of spring used in the dumbbell to connect the two beads.

A quantity which characterizes the structure of the molecule is the second order tensor, $\langle \boldsymbol{QQ} \rangle$, which is known as the conformation tensor, $\boldsymbol{M}$.

$$\boldsymbol{M} = \langle \boldsymbol{QQ} \rangle \tag{5}$$

Here the angular brackets represent an ensemble average.

An equation for $\boldsymbol{M}$ can be obtained from the general diffusion equation [2]:

$$\frac{d\boldsymbol{M}}{dt} - \{\kappa \cdot \boldsymbol{M}\} - \{\boldsymbol{M} \cdot \kappa^T\} = \boldsymbol{M_{(1)}} = \frac{4k_BT}{\zeta}\boldsymbol{\delta} - \frac{4}{\zeta}\langle \boldsymbol{Q}F^c \rangle \tag{6}$$

where, d/dt represents the material derivative operator,

$$\frac{d}{dt} = \frac{\partial}{\partial t} + \boldsymbol{v} \cdot \boldsymbol{\nabla} \tag{7}$$

Dumbbell models can be broadly classified as constant friction and variable friction models. In constant friction models, the frictional drag coefficient (ζ) is assumed to be constant throughout the flow, whereas that in variable friction models is assumed to be a function of polymer conformation [2]. The constant friction models can include either a linear spring connector (Hookean) or a non-linear spring connector (non-Hookean). All variable friction models have non-Hookean connectors. Also, the variable friction models can be further classified into two types. The classical version where self-concentration effects are not included [3] and a new model which has been proposed recently in which ζ is a function of both conformation of polymer molecules and instantaneous pervaded volume fraction of the solution [4, 5].

Constant friction, Hookean dumbbells: Oldroyd-B model

Up to this time point the theory has been developed for dumbbells with any kind of elastic connector, linear or nonlinear. In this section we specialize to the Hookean spring connector for which the function $f(\boldsymbol{Q}) = 1$.

Thus the entropic force for Hookean springs is,

$$\boldsymbol{F^c} = H\boldsymbol{Q} \tag{8}$$

The corresponding equation for polymer stress for Hookean dumbbells,

$$\boldsymbol{\tau_p} = nk_BT\,\boldsymbol{\delta} - n\langle H\,\boldsymbol{Q}\,\boldsymbol{Q} \rangle \tag{9}$$

$$= nk_BT\left(\boldsymbol{\delta} - \frac{H\,\boldsymbol{M}}{k_B\,T}\right) \tag{10}$$

Similarly, the corresponding equation for polymer conformation tensor $\boldsymbol{M}$,

$$\boldsymbol{M_{(1)}} = \frac{4k_BT}{\zeta}\boldsymbol{\delta} - \frac{4}{\zeta}\langle H\,\boldsymbol{Q}\,\boldsymbol{Q} \rangle \tag{11}$$

$$= \frac{4k_BT}{\zeta}\left(\boldsymbol{\delta} - \frac{H\,\boldsymbol{M}}{k_B\,T}\right) \tag{12}$$

The equation above for the equivalent to that in Hookean dumbbell model is the Oldroyd-B model.

Constant friction, non-Hookean dumbells: FENE-P model

The Hookean dumbbell permits infinite extension, however it is known that real molecules cannot be extended infinitely. Thus a non-linear spring with the following force law was presented,

$$\boldsymbol{F^c} = \frac{H\boldsymbol{Q}}{1 - Q^2/L^2} \tag{13}$$

The above force law permits linear (Hookean) behavior for small extensions but gets stiffer as the spring is extended. Also, it restricts the spring extension to a maximum L. This finitely extensible nonlinear elastic (FENE) spring was first presented by [6]. The dumbbell model with the Warner force law is said to be the FENE model.

The corresponding equation for polymer stress for non-Hookean FENE dumbbells,

$$\boldsymbol{\tau_p} = nk_BT\,\boldsymbol{\delta} - n\left\langle \frac{H\boldsymbol{QQ}}{1 - (\boldsymbol{Q}/L)^2} \right\rangle \tag{14}$$

The added non-linearity in the spring force makes it difficult to obtain a closed-form constitutive equation for the polymer stress without making an approximation. A well known approximation is where the denominator in the FENE expression for the connector force is replaced by its ensemble averaged value, i.e.

$$\boldsymbol{F^c} = \frac{H\boldsymbol{Q}}{1 - \langle \boldsymbol{Q} \rangle^2/L^2} = \frac{H\boldsymbol{Q}}{1 - tr\,\boldsymbol{M}/L^2} \tag{15}$$

This pre-averaging is known as the Peterlin approximation and the resulting model as the FENE-P model. The connector force $\boldsymbol{F^c}$ as defined previously can also be written as,

$$\boldsymbol{F^c} = H\boldsymbol{Q}f(\boldsymbol{Q}) \quad \text{and for FENE-P model} \quad f(\boldsymbol{Q}) = \frac{1}{1 - \langle Q^2 \rangle/L^2} \tag{16}$$

After substituting the approximate $\boldsymbol{F^c}$ from Eq. (16) into the expression for polymer stress, Eq. (14),

$$\boldsymbol{\tau_p} = nk_BT\,\boldsymbol{\delta} - nHf\boldsymbol{M} \tag{17}$$

The corresponding expression for conformation tensor ($\boldsymbol{M}$) with the Peterlin approximation,

$$\boldsymbol{M_{(1)}} = \frac{4H}{\zeta}\left[\frac{k_BT}{H}\boldsymbol{\delta} - f\boldsymbol{M}\right] \tag{18}$$

At equilibrium polymer stress $\boldsymbol{\tau_p}$ is equal to zero. Therefore

$$0 = nk_BT\,\boldsymbol{\delta} - nHf_0\boldsymbol{M_0} \tag{19}$$

or

$$f_0\boldsymbol{M_0} = \frac{k_BT}{H}\boldsymbol{\delta} \tag{20}$$

Substituting for k_BT/H in Eq. (18) above

$$\boldsymbol{M_{(1)}} = \frac{4H}{\zeta}\left[\frac{k_BT}{H}\boldsymbol{\delta} - f\boldsymbol{M}\right] = -\frac{4H}{\zeta}\left[f\boldsymbol{M} - f_0\boldsymbol{M}_0\right] \tag{21}$$

At equilibrium the conformation tensor is isotropic and,

$$\boldsymbol{M_0} = \frac{\langle Q^2\rangle_0}{3}\boldsymbol{\delta} \tag{22}$$

where, $\langle Q^2\rangle_0$ is the mean squared end-to-end distance at equilibrium. Therefore we get,

$$\boldsymbol{M_{(1)}} = -\frac{4H}{\zeta}\left[f\boldsymbol{M} - f_0\frac{\langle Q^2\rangle_0}{3}\boldsymbol{\delta}\right] \tag{23}$$

Also,

$$\frac{4H}{\zeta} = \frac{4k_BT}{\zeta}\cdot\frac{H}{k_BT} = \frac{12k_BT}{\zeta\,\langle Q^2\rangle_0}\cdot\frac{1}{f_0} = \frac{1}{\lambda_0}\,\frac{1}{f_0} \tag{24}$$

Here,

$\lambda_0 = \zeta\,\langle Q^2\rangle_0/12k_BT$ represents the polymer relaxation time.

Substituting result from Eq. (24) into Eq. (23),

$$\boldsymbol{M_{(1)}} = -\frac{1}{\lambda_0}\left[\bar{f}\,\boldsymbol{M} - \frac{\langle Q^2\rangle_0}{3}\boldsymbol{\delta}\right] \tag{25}$$

where, $\bar{f} = f/f_0 = \left(1 - \langle Q^2\rangle_0/L^2\right)/\left(1 - \langle Q^2\rangle/L^2\right)$

Now coming to the polymer stress expression, substituting Eq. (20) into Eq. (17), we obtain

$$\boldsymbol{\tau_p} = \frac{3nk_BT}{\langle Q^2\rangle_0}\left[\frac{\langle Q^2\rangle_0}{3}\boldsymbol{\delta} - \bar{f}\boldsymbol{M}\right] \tag{26}$$

Excluded volume interaction

Excluded volume interactions are always present in a polymer molecule, but their effect is controlled by disaffinity to the solvent, which is temperature dependent. At a critical temperature known as the theta temperature (T_θ), excluded volume interactions are exactly balanced by the solvent disaffinity, and

the equilibrium size of the molecule is the same as that of an ideal phantom chain $\langle Q^2 \rangle_0^\theta$ [7]. At temperatures higher than T_θ, $\langle Q^2 \rangle_0 > \langle Q^2 \rangle_0^\theta$ and their ratio is defined as the swelling ratio,

$$\alpha = \left[\frac{\langle Q^2 \rangle_0}{\langle Q^2 \rangle_0^\theta} \right]^{1/2} \tag{27}$$

Here we note that $\langle Q^2 \rangle_0^\theta = b_k\, L$, where b_k is the length of a single Kuhn segment in a polymer chain. The total number of Kuhn segments in any chain is

$$N_k = \frac{L^2}{\langle Q^2 \rangle_0^\theta} \tag{28}$$

and thus $\langle Q^2 \rangle_0^\theta = b_k^2\, N_k$, and $L = b_k\, N_k$. The Kuhn length b_k depends on the monomer chemistry, while L and thus N_k are proportional to the molecular weight of the polymer [7].

The temperature and molecular weight dependence of α is expressed in terms of the solvent quality parameter [8, 9].

$$Z = \frac{v_0}{b_k^3} \left(1 - \frac{T_\theta}{T} \right) \sqrt{N_k} \tag{29}$$

which v_0 is the excluded-volume of a single Kuhn segment and is determined by the chemistry of the monomer and solvent. Since, v_0, b_k and T_θ are independent of the molecular weight, the equation above implies that at the same temperature and polymer-solvent chemistry, the solvent quantities of two different molecular weight samples are related as

$$\frac{Z_1}{Z_2} = \frac{\sqrt{N_{k,1}}}{\sqrt{N_{k,2}}} \tag{30}$$

Hence if the solvent quality Z_{ref} is known for a reference molecular weight corresponding to $N_{k,ref}$, then the solvent quality for any other molecular weight sample is calculated as,

$$Z = Z_{ref} \sqrt{\frac{N_k}{N_{k,ref}}} \tag{31}$$

Given Z, the swelling ratio is given by an empirical fir through experimental and molecular simulation results [9]:

$$\alpha^2(Z) = [1 + 9.8Z + 14Z^2 + 32Z^3]^{0.13} \tag{32}$$

For later use, we note here that the discussion above implies that the swelling of a chain is non-uniform; if Z is the solvent quality corresponding to a chain of N_k segments, then the solvent quality corresponding to a sub-chain consisting of $N_k' < N_k$ segments is $Z' = Z\sqrt{N_k'/N_k}$, and the mean squared equilibrium size of the sub-chain is

$$\xi^2 = b_k^2 N_k' \alpha^2(Z') \tag{33}$$

Variable friction dumbbell models

The polymer relaxation time ($\lambda_0 = \zeta_0 \langle Q^2\rangle_0/12k_BT$) that has been used in the FENE-P model assumes a constant friction coefficient (ζ_0). λ_0 is measured typically by using small-amplitude oscillatory shear (SAOS) rheometry for a given solution at a given concentration. If $\langle Q^2\rangle_0$ is also known at that same concentration then the above equation can be used to estimate ζ_0. This theory is valid under quiescent conditions. However in extensional flows, when the polymer coils unravel and stretch, the friction coefficient also changes along with this change in conformation of the polymer. We assume, ζ is the new friction coefficient for partially stretched molecules. The corresponding polymer relaxation time is,

$$\lambda = \zeta \langle Q^2\rangle_0/12k_BT \tag{34}$$

and hence,

$$\lambda(\boldsymbol{M}, c) = \lambda_0 \left[\frac{\zeta(\boldsymbol{M}, c)}{\zeta_0}\right] \tag{35}$$

Thus equation (25) for the FENE-P model is modified as,

$$\boldsymbol{M}_{(1)} = -\frac{1}{\lambda}\left[\bar{f}\,\boldsymbol{M} - \frac{\langle Q^2\rangle_0}{3}\boldsymbol{\delta}\right] = -\frac{1}{\lambda_0}\frac{1}{\zeta/\zeta_0}\left[\bar{f}\,\boldsymbol{M} - \frac{\langle Q^2\rangle_0}{3}\boldsymbol{\delta}\right] \tag{36}$$

Variable friction coefficient In an extensional flow, as the molecules stretch, the polymer conformation changes. Thus the drag coefficient ζ of the molecules is no longer equal to the mean friction coefficient of isotropic coiled polymer molecules, ζ_0.

However because a general molecular theory is still not available, ζ is expected to lie somewhere between ζ_0 and an estimated friction coefficient ζ_r, that is derived assuming that partially stretched chains are slender rod-like objects of length $l = M_{zz}^{1/2}$ and diameter $d = M_{rr}^{1/2}$, all aligned in the principal stretching direction.

Hence ζ is approximated by a simple "mixing rule" between ζ_0 and ζ_r,

$$\zeta = \zeta_0(1-\chi) + \zeta_r(l, d)\,\chi \tag{37}$$

where,

$$\chi = \frac{l - l_0}{L - l_0} = \frac{M_{zz}^{1/2} - l_0}{L - l_0} \tag{38}$$

where, $l_0 = M_{zz,0}^{1/2} = M_{rr,0}^{1/2} = \langle Q^2\rangle_0^{1/2}/\sqrt{3}$

Hence,

$$\frac{\zeta}{\zeta_0} = (1-\chi) + \frac{\zeta_r(l, d)}{\zeta_0}\chi \tag{39}$$

Note that $\zeta_0 \neq \zeta_Z$, the equilibrium friction coefficient of isolated polymers (the "Zimm" drag coefficient) and only approaches ζ_Z as $c/c^* = \phi_0 \to 0$. However, $\zeta_0/\zeta_Z = \lambda_0/\lambda_z$ and the ratio ζ_0/ζ_Z can be obtained from SAOS measurements.

Typical estimates of ζ_r are derived in terms of the ratio ζ_r/ζ_Z. Hence, Eq. (39) is better written as,

$$\frac{\zeta}{\zeta_0} = (1-\chi) + \left(\frac{\zeta_r(l,d)/\zeta_Z}{\zeta_0/\zeta_Z}\right)\chi \tag{40}$$

Thus the modeling task is to get the values of $\zeta_r(l,d)/\zeta_Z$ to calculate ζ/ζ_0, which can then be used in governing equation, Eq. (36).

Conformation Dependent Drag with self concentration: CDD-sc

Polymer concentration typically understood in terms of c/c^* in literature is actually a volume fraction, since

$$c \sim n \tag{41}$$

$$c^* \sim 1/V_0 \tag{42}$$

where, V_0 is the average equilibrium volume of a molecule.

Hence,

$$\frac{c}{c^*} = n\,V_0 \tag{43}$$

We use a notation that is standard in suspension literature for the volume fraction and denote the equilibrium volume fraction as $\phi_0 = (c/c^*)$.
For molecules that stretch and deform, an estimate of the volume pervaded by the molecular coils comes from M. Average coil volume $= V = \sqrt{M_1\,M_2\,M_3}$, where M_i's are the eigen values of M. For uniaxial extensional flows, the average coil volume $= V = \sqrt{M_{zz}\,M_{rr}^2}$. In other words, the average coil shape is pictured as a cylinder, Therefore the instantaneous volume fraction of a solution is, $\phi = n\,l\,d^2$

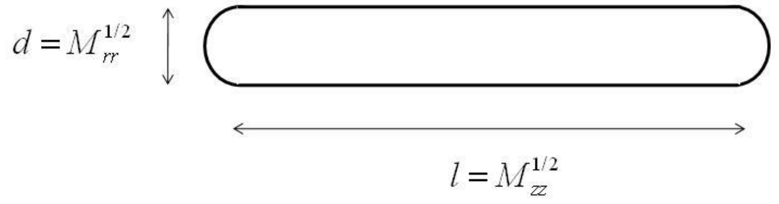

Figure 1: Average coil shape is pictured as a cylinder

Since for a given solution, n remains unchanged, we get,

$$n = \frac{\phi}{l\,d^2} = \frac{\phi_0}{l_0\,d_0^2} \tag{44}$$

Therefore using Eq. (44) it is possible to calculate ϕ at any given time provided the initial ϕ_0 and the instantaneous l and d from $\boldsymbol{M}$ are known.

$$\phi = \phi_0 \frac{l\, d^2}{l_0\, d_0^2} \tag{45}$$

Since polymers can inter-penetrate, ϕ can be greater than 1. Further even if $\phi_0 << 1$, ϕ could exceed 1 if instantaneous volume $l\, d^2$ exceeds $l_0\, d_0^2$ significantly. Evidence for such "self-concentration" comes from Brownian Dynamics simulations of polymer solutions [10, 11].

Intermolecular separation in extensional flows: Consider a solution with all molecules aligned in the stretching direction. The average separation in the transverse direction between center lines h, is such that

$$n\, l\, h^2 = 1 \tag{46}$$

or

$$\frac{h}{d} = \frac{1}{\sqrt{n\, l\, d^2}} = \frac{1}{\sqrt{\phi}} \tag{47}$$

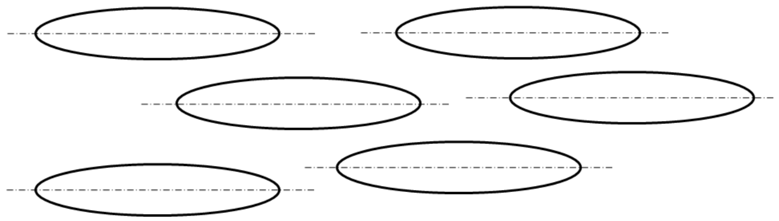

Figure 2: Individual polymer molecules are aligned in the direction of flow

Hence as $\phi \to 0$, $h/d \to \infty$ and molecules are far apart. But as $\phi \to 1$, $h \to d$ and when $\phi > 1$, $h < d$ and molecules overlap transversely.

For the purpose of rescaling variables, it is useful to see that,

$$\frac{h}{d_0} = \frac{1}{\sqrt{n\, l_0\, d_0^2 (l/l_0)}} = \frac{1}{\sqrt{\phi_0 (l/l_0)}} \tag{48}$$

As the polymer molecules start stretching, the value of instantaneous pervaded volume fraction is calculated using Eq. (45). Based on ϕ and h, we can identify three main dilution regimes. The expression for ζ_r/ζ_Z in each of these regimes is discussed below:

Dilute regime, $\phi \leqslant 0.01$

For this regime, [12]'s interpolation formula for non-dilute rod suspensions is used to derive an expression for ζ, suggestive of a solution with partially stretched but non-overlapping chains.

$$\frac{\zeta_r}{\zeta_Z} = \left[\frac{K/\sqrt{3}}{K/\sqrt{3} + \ln(F)} \right] \frac{l}{l_0} \tag{49}$$

where,

$$F = \frac{l/d}{1 + l/h}, \qquad \text{when} \qquad l/d > 1 + l/h \tag{50}$$

$$= 1 \qquad \text{otherwise} \tag{51}$$

This method is used to ensure that ζ always increases for $l/d > 1$. Although Eq. (50) is asymptotically valid for large aspect ratios, at small aspect ratios just above 1, ζ_r/ζ_Z first decreases with Eq. (50) with increasing l/d, before becoming an increasing function of l/d. To avoid this unphysical behavior, Eq. (51) is used. Effectively the log term is in use for all aspect ratios above 1.11, so the adjustment by Eq. (51) occurs only near a very small domain of aspect ratios.

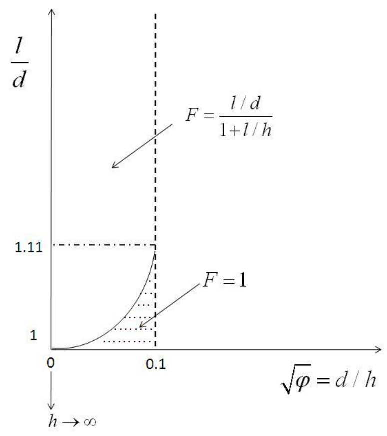

Figure 3: Graph showing two distinct regions of the dilute regime

Intermediate regime, $0.01 < \phi < 1$

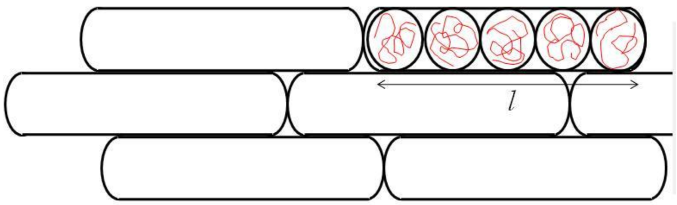

Figure 4: Dynamic critical overlap, $\phi = 1$

No simple expressions are available for the drag coefficient in this regime as the inter-molecular HI become increasingly important as molecules approach overlap. But it is expected that screening of hydrodynamic interactions sets in at incipient overlap at $\phi = 1$ when partially stretched coils just begin to

overlap. At this condition the partially stretched coil is modeled as a string of $N_b = l/d$ "beads" of diameter d. Intramolecular hydrodynamic interactions (HI) persist within each bead, but the presence of the neighboring molecules dampens velocity perturbations at length scales larger than d, that is there are no HI across segments contained in different beads. Hence the friction coefficient of the whole chain is the Rouse-like friction of N_b beads, i.e.

$$\zeta_r = N_b \zeta_d, \qquad (\zeta_d \text{ is the friction of each bead}) \tag{52}$$

but Zimm-like HI within each bead gives,

$$\zeta_d = \frac{d}{d_0}\zeta_Z \tag{53}$$

Hence when $\phi = 1$,

$$\frac{\zeta_r}{\zeta_Z} = \frac{l}{d}\frac{d}{d_0} = \frac{l}{d_0} = \frac{l}{l_0} \tag{54}$$

When $0.01 < \phi < 1$, for any l/d, we interpolate linearly w.r.t. ϕ the value predicted at $\phi = 0.01$ and the value predicted at the same l/d at $\phi = 1$. The final expression for ζ_r/ζ_Z is given as,

$$\frac{\zeta_r}{\zeta_Z} = \left[\frac{K/\sqrt{3}}{K/\sqrt{3} + \ln(F_c)}\right]\left(\frac{l}{l_0}\right)\left(\frac{1-\phi}{1-0.01}\right) + \left(\frac{l}{l_0}\right)\left(\frac{\phi - 0.01}{1-0.01}\right) \tag{55}$$

where,

$$F_c = \frac{l/d}{1 + 0.1\, l/d}, \qquad \text{for} \quad \frac{l}{d} \geqslant \frac{1}{0.9} \tag{56}$$

$$= 1 \qquad \text{for} \quad \frac{l}{d} < \frac{1}{0.9} \tag{57}$$

Semi dilute overlapping regime: $\phi \geqslant 1$

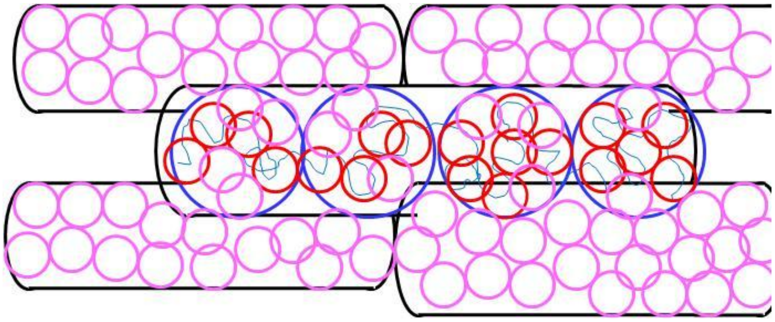

Figure 5: Semi-dilute regime with overlapping polymer molecules

In this regime the solution is no longer dilute and the adjacent polymer molecules begin overlapping each other. To calculate the friction coefficient in this case, the concept of "blobs" is used [7, 8]. A blob represents the length scale at which segments of any molecule encounter segments from other chains. Again each molecule can be considered to consist of $N_b = l/d$ "beads". As shown in Figure 5, each bead (blue circles) contains blobs and each blob contains polymer segments. The red circles indicate blobs of a particular chain whose mean drag needs to be calculated. The pink circles are blobs belonging to other chains. Intramolecular HI i.e. Zimm behavior is only restricted to within a blob and no HI takes place (i.e. Rouse-like behavior) between blobs of a particular chain. The blobs exactly fill the full space i.e. total volume of all blobs from all chains is equal to the solution volume. This means that if ξ is the size of a single blob, and each chain has N_ξ blobs, then

$$n\,N_\xi\,\xi^3 = 1$$

or

$$\phi N_\xi\,\xi^3 = ld^2 \tag{58}$$

The size of each blob is determined by the fact that within each blob, an equilibrium like structure persists and

$$\xi^2 = b'_k\,L_\xi\,\alpha^2(z_\xi) = b'_k\,b_k\left(\frac{N_k}{N_\xi}\right)\alpha^2(z_\xi) \tag{59}$$

where b'_k is a modified Kuhn-segment length, due to local stretching by the flow and z_ξ is the solvent quality of the sub-chain of contour length L_ξ contained within the blob such that,

$$z_\xi = \frac{z}{\sqrt{N_\xi}}\sqrt{N_k/N_\xi} = \frac{z}{\sqrt{N_\xi}} \tag{60}$$

b'_k is eliminated from the above equations by assuming that d is similarly related,

$$d^2 = b'_k b_k\left(\frac{N_k}{N_b}\right)\alpha^2(z_b) \tag{61}$$

where

$$z_b = z\sqrt{\frac{N_k/N_b}{N_b}} = \frac{z}{\sqrt{N_b}} \tag{62}$$

From Eqs. (58-62) above, z and ϕ_0 are specified as input parameters and at any instant we have l and d from the ODE solutions. ϕ is also known from ϕ_0, l and d according to Eq. (44). So the unknown quantities are ξ, N_ξ and b'_k. Out of these ξ and N_ξ are important. So we divide Eq. (59) by (61), to eliminate b'_k and get

$$\frac{\xi^2}{d^2} = \frac{N_b}{N_\xi}\,\frac{\alpha^2(z_\xi)}{\alpha^2(z_b)} \tag{63}$$

Then Eqs. (58) and (63) are combined to eliminate ξ by first taking Eq. (63) to the power of 3/2,

$$\frac{\xi^3}{d^3} = \left(\frac{N_b}{N_\xi}\right) \frac{\alpha^3(z_\xi)}{\alpha^3(z_b)}$$

and divide by Eq. (58) to get rid of ξ^3 and get:

$$\frac{N_\xi}{N_b} = \phi^2 \frac{\alpha^6(z_\xi)}{\alpha^6(z_b)} \tag{64}$$

But $N_\xi = (z/z_\xi)^2$ and $N_b = (z/z_b)^2$ from Eqs. (60) and (62) and $l/d = N_b$; hence

$$z_\xi^2\,\alpha^6(z_\xi) = \frac{z_b^2\,\alpha^6(z_b)}{\phi^2} = \frac{z_b^2\,\alpha^6(z_b) d_0^3}{\phi_0\, l\, d^2} \tag{65}$$

All quantities on the R.H.S. are known, and we must solve the function for Eq. (64) after using the function in Eq. (32) with z_ξ as the variable. From z_ξ, N_ξ can be calculated using Eq. (60).

Then the drag coefficient is calculated as the Rouse drag of N_ξ blobs

$$\zeta_r = N_\xi\, \zeta_\xi \tag{66}$$

and ζ_ξ is the Zimm-drag of a single blob

$$\zeta_\xi = \left(\frac{\xi}{d_0}\right) \zeta_Z \tag{67}$$

or

$$\begin{aligned}
\frac{\zeta_r}{\zeta_Z} &= \frac{N_\xi\, \xi}{d_0} \\
&= \frac{l\, d^2}{\phi\, \xi^2\, d_0} \qquad\qquad \text{from Eq. 58} \\
&= \frac{1}{\phi}\, \frac{d^2}{\xi^2}\, \frac{l}{d_0} \\
&= \frac{1}{\phi}\, \frac{l}{d_0}\, \frac{N_\xi}{N_b}\, \frac{\alpha^2(z_\xi)}{\alpha^2(z_b)} \\
&= \frac{1}{\phi}\, \frac{l}{d_0}\, \phi^2\, \frac{\alpha^2(z_\xi}{\alpha^2(z_b)}\, \frac{\alpha^2(z_\xi)}{\alpha^2(z_b)} \\
\frac{\zeta_r}{\zeta_Z} &= \phi\, \frac{\alpha^4(z_\xi)}{\alpha^4(z_b)}\, \frac{l}{d_0} = \phi\, \frac{\alpha^4(z_\xi)}{\alpha^4(z_b)}\, \frac{l^2\, d^2}{d_0^4}
\end{aligned} \tag{68}$$

Thus given ϕ_0 at any instant, if l and d are known, z_ξ can be calculated by solving Eq. (65) and subsequently, ζ_r/ζ_Z can be calculated.

This model is referred to as the "Conformation-Dependent Drag with self concentration model" or CDD-sc model.

Standard FENE-P model can be obtained by ignoring the concentration and conformation dependence of the frictional drag in the CDD-sc model.

References

[1] R. B. Bird, R. C. Armstrong, and O. Hassager. *Dynamics of Polymeric Liquids. Volume 1: Fluid Mechanics.* Wiley Intersci., New York, 1987.

[2] R. B. Bird, C. F. Curtiss, R. C. Armstrong, and O. Hassager. *Dynamics of Polymeric Liquids. Volume 2: Kinetic Theory.* Wiley Intersci., New York, 1987.

[3] P.G. De Gennes. Coil-stretch transition of dilute flexible polymers under ultrahigh velocity gradients. *J. Chem. Phys.*, 60:5030–5042, 1974.

[4] R. Prabhakar. Predicting capillary thinning of dilute polymer solution filaments with an improved variable-friction model for polymeric stresses. In *Proc. CHEMECA 2011: Engineering a Better World, 2543–2552*, Sydney, Australia, 18-21 September 2011.

[5] R. Prabhakar. Enhancement of coil-stretch hysteresis by self-concentration in polymer solutions. arXiv:1209.0163 [cond-mat.soft], 2012.

[6] H.R. Warner. Kinetic theory and rheology of dilute suspensions of finitely extendible dumbbells. *Ind. Eng. Chem. Fundam.*, 11(3):379–387, 1972.

[7] M. Doi and S.F. Edwards. *The theory of polymer dynamics.* Calendron Press, Oxford, Oxford, 1986.

[8] M. Rubinstein and R. H. Colby. *Polymer Physics.* Oxford University Press, Oxford, 2003.

[9] K. S. Kumar and J. R. Prakash. Equilibrium swelling and universal ratios in dilute polymer solutions: Exact brownian dynamics simulations for a delta function excluded volume potential. *Macromol.*, 36:7842–7856, 2003.

[10] R. Prabhakar. *Predicting the Rheological Properties of Dilute Polymer Solutions Using Bead-spring Models: Brownian Dynamics Simulations and Closure Approximations.* PhD thesis, Monash University, 2005.

[11] C. Stoltz, J.J. De Pablo, and M.D. Graham. Concentration dependence of shear and extensional rheology of polymer solutions: Brownian dynamics simulations. *J. Rheol.*, 50(2):137–167, 2006.

[12] G. K. Batchelor. The stress generated in a non-dilute suspension of elongated particles by pure straining motion. *J. Fluid Mech.*, 46:813–829, 1971.
