# Supplementary material for: Influence of Polymer Relaxation Time on the Electrospinning Process: Numerical Investigation
Source: Polymers (Basel). 2017 Oct 12;9(10):501. doi: 10.3390/polym9100501 (PMC6418928; doi:10.3390/polym9100501)
Supplement: Supplementary file 1 [file polymers-09-00501-s001.zip › supportinginfo2.pdf]

Influence of polymer relaxation time on the electrospinning process: numerical investigation

Siddharth Gadkari

Supplementary Material 2: Validation Studies

1 Validation studies

The numerical analysis was validated by comparing against previously reported results in literature. For the Newtonian case ($B = 1$ or $\phi_0 = 0$) an excellent agreement was obtained with the results of [1] and [2] as shown in Figures 1 and 2.

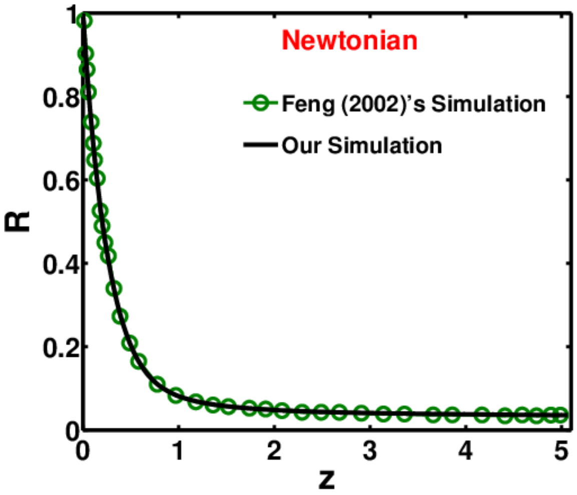

Figure 1: Comparison between thinning profile predictions of our simulation for Newtonian jets to that obtained by [1] using the following parameter values: Re $= 4.451 \times 10^{-3}$, Fr $= 8.755 \times 10^{-3}$, We $= 1.099 \times 10^{-3}$, Pe $= 1.835 \times 10^{-4}$, ϵ_E $= 0.7311$, $\beta = 45.5$, $\chi = 75$, $\Omega = 5.914$.

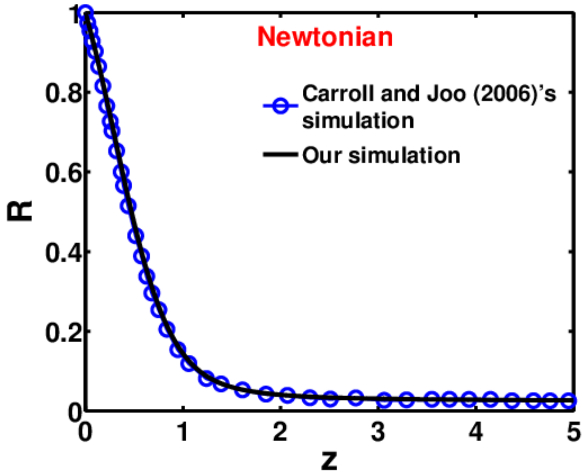

Figure 2: Comparison between thinning profile predictions of our simulation for Newtonian jets to that obtained by [2] using the following parameter values: Re $= 9.00 \times 10^{-4}$, Fr $= 9.18 \times 10^{-4}$, We $= 4.43 \times 10^{-3}$, Pe $= 6.53 \times 10^{-5}$, $\epsilon_E = 1.469$, $\beta = 41.5$, $\chi = 327$, $\Omega = 2.29$.

Similarly, for polymer solutions, Giesekus and Oldroyd-B constitutive models were implemented in the current study and the results were compared with those reported by [3] and [2] respectively. As can be clearly seen from the figures 3 and 4, even for the non-Newtonian case an excellent agreement was found.

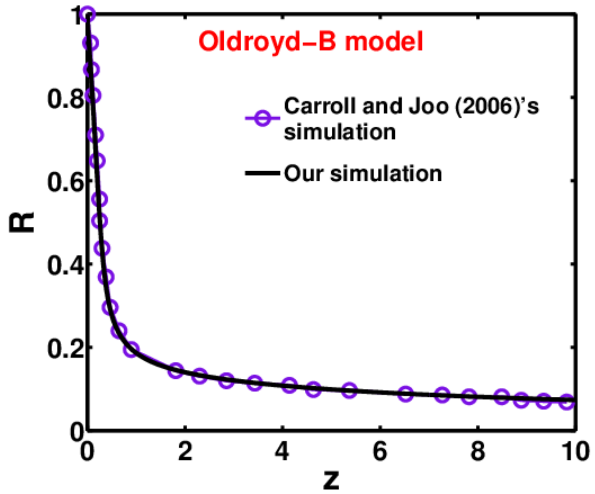

Figure 3: Comparison between thinning profile predictions of our simulation using Giesekus model to that obtained by [3] using the following parameter values: $\mathrm{Re} = 2.5 \times 10^{-3}$, $\mathrm{Fr} = 0.1$, $\mathrm{We} = 0.1$, $\mathrm{Pe} = 0.1$, $\epsilon_E = 1$, $\beta = 40$, $\chi = 600$, $\Omega = 0.1$, $\mathrm{De} = 10$, $\alpha = 0.01$.

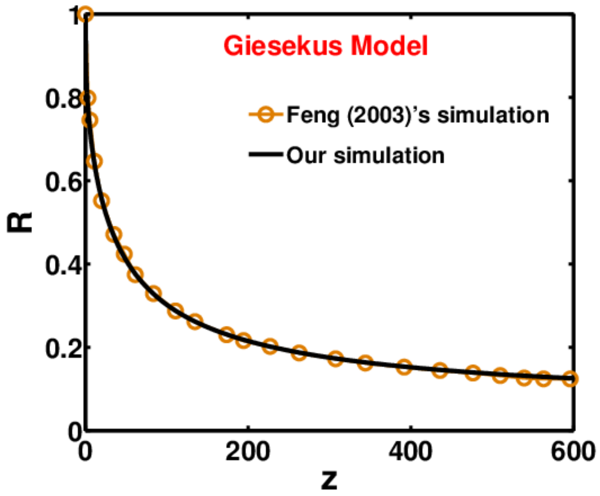

Figure 4: Comparison between thinning profile predictions of our simulation using Oldroyd-B model to that obtained by [2] using the following parameter values: Re $= 5.15 \times 10^{-3}$, Fr $= 9.18 \times 10^{-4}$, We $= 1.31 \times 10^{-4}$, Pe $= 2.5 \times 10^{-3}$, $\epsilon_E = 10.6$, $\beta = 2$, $\chi = 270$, $\Omega = 1.42$, De $= 2.65 \times 10^{-2}$

References

[1] J.J. Feng. The stretching of an electrified non-newtonian jet: A model for electrospinning. *Phys. Fluids*, 14(11):3912–3926, 2002.

[2] C.P. Carroll and Y.L. Joo. Electrospinning of viscoelastic boger fluids: Modeling and experiments. *Phys. Fluids*, 18(5), 2006.

[3] J.J. Feng. Stretching of a straight electrically charged viscoelastic jet. *J. Non-Newtonian Fluid Mech.*, 116(1):55–70, 2003.
